# Fumarate hydratase: a new checkpoint of metabolic regulation in inflammatory macrophages

**DOI:** 10.1038/s41392-023-01594-2

**Published:** 2023-09-04

**Authors:** Aicha Jeridi, Theodore S. Kapellos, Ali Önder Yildirim

**Affiliations:** 1 Comprehensive Pneumology Center (CPC), Institute of Lung Health and Immunity (LHI), Member of the German Center for Lung Research (DZL), Helmholtz Munich, Munich, Germany; 2 Institute of Experimental Pneumology, University Hospital, Ludwig-Maximilians University, München, Germany

**Keywords:** Translational research, Innate immunity

A recent article published by Hooftman et al.^1^ in Nature identified a crucial function for fumarate hydratase (*FH*) in bone marrow-derived macrophages (BMDMs). Pharmacological inhibition or genetic ablation of FH led to increased fumarate levels and downstream suppression of mitochondrial respiration implying the importance of cellular metabolic states and cytokine balance shift in innate immunity.

The impact of immunometabolic reprogramming has gained increasing attention as a key regulating factor for homeostasis, activation, proliferation and differentiation of immune cells over recent years.^2,3^ Macrophages (MΦs) respond to signals they receive from other cells and their microenvironment, which causes metabolic remodeling, resulting in changes to their function and activation status. This high degree of plasticity allows them to quickly adapt to a constantly changing environment. As a response to inflammation in autoimmune diseases such as systemic lupus erythematosus (SLE) as presented in the present study, or sterile inflammation in other chronic diseases such as COPD and other non-communicable diseases, MΦs undergo a shift in their metabolic state. Different metabolic activities including glycolysis, oxidative phosphorylation, pentose phosphate pathway (PPP) as well as lipid metabolism^2^ have been shown to modulate and regulate MΦ function in both inflammation and resolution. Crucially, TCA cycle intermediate metabolites are now well accepted as inflammatory regulators in LPS-treated MΦs; succinate acts in both a paracrine and autocrine manner and stimulates the secretion of IL-1β, IL-1ra and IL-10, chemotaxis and production of the anti-inflammatory prostaglandin PGE2. α-ketoglutarate supports MΦ polarization to IL-4 and endotoxin tolerance, fumarate promotes the production of TNF and IL-6 upon restimulation with LPS via histone demethylation inhibition. Furthermore, citrate efflux from the mitochondria is associated with ROS, NO and prostaglandin production, whereas itaconate has been shown to bear antibacterial and anti-inflammatory capacity via the reduction of IL-6, IL-1β and IL-12p40 levels. In a very recent study, we confirmed the upregulation of *Alox5* in pro-inflammatory MΦs, revealing a new and unique role for Alox5 in producing leukotriene B4 (LTB4) as a driver of ferroptotic cell death in ATII cells in both COPD patients and animal models.^4^

MΦs were originally classified by a range of activation states; classically activated and alternatively activated MΦs. Lipopolysaccharide (LPS) or cytokines such as IFN-γ and TNF-α induce a pro-inflammatory state—referred to as “M1” MΦs; Interleukin IL-4 and IL-13 result in- “M2” MΦs. However, the advent of transcriptomics revealed that MΦs undertake diverse phenotypes that lie within a spectrum of the M1 to M2 dichotomy and are characterized by distinct gene signatures. For example, MΦs stimulated with LPS are transcriptomically distinguished from TNF/PGE_2_/P3C-primed MΦs, similarly for IL-4 and fatty acid-challenged MΦs. A new consensus has therefore been met where MΦ activation states are defined by the stimulant used in the in vitro/ex vivo microenvironment. Distinct metabolic pathways have been associated with MΦ activation states. Pro-inflammatory MΦs rely on glycolysis, a process known as “the Warburg effect”. First, it was observed by Hard et al.,^5^ pro-inflammatory MΦs decrease oxidative phosphorylation and increase glycolysis, even more during phagocytosis with almost no oxygen consumption. In order to increase glycolytic activity, pro-inflammatory MΦs upregulate *GLUT1*. In addition, pro-inflammatory MΦs make use of PPP, the main source of NADPH, which protects them against oxidative stress and drives ROS production to kill pathogens. Importantly, along with the activation of the glycolysis pathway, MΦs experience a robust shutdown of oxidative metabolism and disruption of the TCA cycle. The interruption occurs at two levels: The inhibition of succinate dehydrogenase, which causes the accumulation of succinate leading to succinylation of many proteins resulting in the stabilization of the transcription factor HIF-1α that is required for cytokine expression. Another breakdown happens at the level of isocitrate dehydrogenase, which results in the accumulation of isocitrate and citrate, components crucial for the synthesis of itaconate. Not only is oxidative metabolism impaired, but LPS also triggers reverse electron transport to promote the production of mitochondrial ROS.

Despite widespread literature on MΦ phenotypic plasticity, the relationship between MΦ metabolic profile and the elicited immune responses during inflammatory conditions remain incompletely understood. Hooftman and colleagues revealed a role for FH in the regulation of MΦ cytokine production upon LPS challenge.^1^ Similarly, FH suppressed B-cell activation and anti-tumor capacity of CD8 T cells in the tumor microenvironment. In an unbiased metabolomics approach, the authors aimed at identifying metabolic changes in BMDMs undergoing acute and prolonged LPS stimulation. Accumulation of fumarate in the cytosol was found to be the result of argininosuccinate cleavage due to the overexpression of argininsuccinate synthase (*Ass1*). Using isotope-assisted tracing, the authors demonstrated that glutamine is partly a source for the aspartate-arginino succinate shunt metabolites and identified glutaminolysis as a general source of carbon in the TCA cycle.

Both treatments with an FH inhibitor, as well as the tamoxifen-induced conditional knockout of *Fh1* in MΦs, showed similar bioenergetic changes and TCA cycle rewiring. The authors observed that FH inhibition, as well as low concentrations of fumarate esters, suppress glycolysis. Mechanistically, RNA-seq analysis revealed that BMDMs suppress IL-10 expression and release upon FH inhibition, whereas TNF production was increased. Finally, to delineate the IFN response, the authors focused on the regulators of *Ifnb1* expression upon FH inhibition and found that Nrf2 restrains IFN response gene transcription. The most exciting result, however, was the link between mitochondrial stress characterized by the release of mitochondrial DNA and RNA and the IFN-β response triggered by TLR7 and MDA5. These findings raise significant questions for future investigation. First, the consequences of altered *FH* expression in pathological conditions remain unexplored. For example, in many non-communicable chronic diseases like COPDi, cells are exposed to altered microenvironments, such as that found in diabetes or highly inflamed conditions where TLR4 is permanently activated (Fig. 1). This should be particularly considered in the light of their differing results achieved under acute and chronic sterile and non-sterile exposures. Second, the FH mode of action in modulating MΦ functionalities through epigenetic imprinting to impact upon immune-metabolic control e.g. by trained immunity should be considered. Lastly, it would be intriguing to study the role of FH, but also other TCA cycle enzymes, in primary human and murine monocyte-derived MΦs that have been recently described with single-cell transcriptomics and assess how location affects FH and cytokine production.

**Fig. 1:**
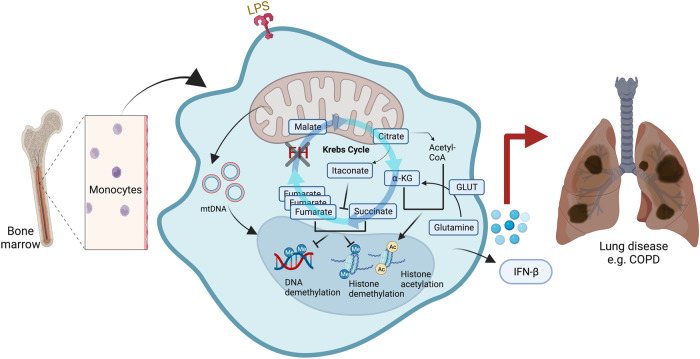
The function of cellular metabolism and epigenetic regulation in macrophages. In response to environmental trigger bone marrow-derived macrophages undergo changes in their activation and function as a result of epigenetic regulation. Alterations in DNA and histones such as methylation or acetylation have a direct impact on the regulation of gene expression shaping polarization, function and fate of monocytes. Changes in the abundance of metabolites such as TCA intermediates and modification of the DNA or histones are tightly linked. Pro-inflammatory macrophages rely on glycolysis and present disruptions in the TCA cycle resulting in the accumulation of itaconate, succinate and fumarate. Monocyte-derived macrophages exhibit changes in gene expression based on metabolic adaptation leading to the secretion of certain cytokines that have a great impact on disease development and progression such as chronic lung diseases. Created with BioRender.com

Together, this exciting work by Hooftman et al.^1^ identified a new mechanism for FH activity and fumarate accumulation that has a direct impact on the inflammatory response and MΦ function. This is particularly relevant to TCA cycle induced mitochondrial stress conditions that result in released mtDNA and mtRNA that result in the activation of IFN signaling.

## References

[1] Hooftman A (2023). Macrophage fumarate hydratase restrains mtRNA-mediated interferon production. Nature.

[2] Liu Y (2021). Metabolic reprogramming in macrophage responses. Biomark. Res..

[3] Sorgi CA (2017). Dormant 5-lipoxygenase in inflammatory macrophages is triggered by exogenous arachidonic acid. Sci. Rep..

[4] Gunes Gunsel G (2022). The arginine methyltransferase PRMT7 promotes extravasation of monocytes resulting in tissue injury in COPD. Nat. Commun..

[5] Hard GC (1970). Some biochemical aspects of the immune macrophage. Br. J. Exp. Pathol..

